# Rapid viral suppression using integrase inhibitors during acute HIV-1 infection

**DOI:** 10.1093/jac/dkae391

**Published:** 2024-11-06

**Authors:** Mehri S McKellar, Jessica R Keys, Lindsey M Filiatreau, Kara S McGee, Joann D Kuruc, Guido Ferrari, David M Margolis, Joseph J Eron, Charles B Hicks, Cynthia L Gay

**Affiliations:** Department of Medicine, Duke University, Durham, NC, USA; Department of Epidemiology, Gillings School of Public Health, University of North Carolina at Chapel Hill, Chapel Hill, NC, USA; Department of Epidemiology, Gillings School of Public Health, University of North Carolina at Chapel Hill, Chapel Hill, NC, USA; Division of Infectious Diseases, School of Medicine, Washington University in St. Louis, St. Louis, MO, USA; Department of Medicine, Duke University, Durham, NC, USA; Department of Medicine, University of North Carolina at Chapel Hill, Chapel Hill, NC, USA; UNC HIV Cure Center, University of North Carolina at Chapel Hill, Chapel Hill, NC, USA; Department of Surgery, Duke University, Durham, NC, USA; Department of Medicine, University of North Carolina at Chapel Hill, Chapel Hill, NC, USA; UNC HIV Cure Center, University of North Carolina at Chapel Hill, Chapel Hill, NC, USA; Department of Medicine, University of North Carolina at Chapel Hill, Chapel Hill, NC, USA; UNC HIV Cure Center, University of North Carolina at Chapel Hill, Chapel Hill, NC, USA; ViiV Healthcare, Research Triangle Park, NC, USA; Department of Medicine, University of North Carolina at Chapel Hill, Chapel Hill, NC, USA; UNC HIV Cure Center, University of North Carolina at Chapel Hill, Chapel Hill, NC, USA

## Abstract

**Background:**

Antiretroviral therapy (ART) is recommended for all individuals with HIV infection, including those with acute HIV-1 infection (AHI). While recommendations are similar to those for chronic infection, efficacy data regarding treatment of acute HIV is limited.

**Methods:**

This was a single arm, 96-week study of a once-daily integrase inhibitor (INSTI)-based regimen using elvitegravir/cobicistat/emtricitabine/tenofovir disoproxil fumarate (EVG/COBI/FTC/TDF) in AHI. Primary endpoint was proportion of participants with HIV-1 RNA <200 copies/mL and <50 copies/mL by treatment weeks 24 and 48, respectively. We also examined time to viral suppression and weight gain after treatment initiation. Outcomes and characteristics were compared with a historical AHI cohort using a non-nucleoside reverse transcriptase inhibitor (NNRTI)-based regimen with efavirenz/emtricitabine/tenofovir disoproxil fumarate (EFV/FTC/TDF).

**Results:**

Thirty-three participants with AHI were enrolled with 31 available for analyses. Most were African American (61%) and men who have sex with men (73%). Median age was 26 (IQR 22–42). Demographics were similar between the two AHI cohorts. By Week 24, 100% in the INSTI and 99% in the NNRTI cohort were <200 copies/mL; by Week 48, 100% in both cohorts were <50 copies/mL. Time to viral suppression was shorter in the INSTI cohort (median 54 versus 99 days). Mean weight change was similar with a 3.6 kg increase in the INSTI cohort and 2.4 kg in the NNRTI cohort at 96 weeks.

**Conclusions:**

INSTI-based ART during AHI resulted in rapid and sustained viral suppression. Over 96 weeks, weight increased in the INSTI-based cohort but was similar to weight increase in a historical NNRTI-based AHI cohort.

## Introduction

Antiretroviral therapy (ART) is recommended for all individuals with HIV infection, including those with acute HIV-1 infection (AHI), defined as the period between HIV-1 acquisition and the development of HIV-1-specific antibodies.^1^ Treatment of individuals during AHI may lower the viral set point,^2^ reduce the size of the HIV-1 reservoir^3,4^ and provide immunologic benefits,^5,6^ in addition to relieving symptoms and preventing transmission to uninfected partners during a period of high viremia.^7^

As with treatment of chronic HIV, the goal of therapy in AHI is to suppress plasma HIV-1 RNA to undetectable levels as quickly and effectively as possible. Presently, there are no specific guidelines on which regimen to use in AHI, other than ensuring that the patient is HLA-B*5701 negative before starting an abacavir-containing regimen. The U.S. Department of Health and Human Services (DHHS) guidelines suggest using an integrase strand transfer inhibitor (INSTI) such as bictegravir (BIC) or dolutegravir (DTG), in combination with tenofovir disoproxil fumarate (TDF) or tenofovir alafenamide (TAF) and emtricitabine (FTC) or lamivudine (3TC), for acute and recent (early) HIV infection if drug resistance testing results are not available before starting treatment.^8^ For persons with a history of using long-acting cabotegravir (CAB-LA) for pre-exposure prophylaxis (PrEP), a pharmacologically boosted protease inhibitor (PI)-based regimen, specifically boosted darunavir, with TDF (or TAF) and FTC (or 3TC) is recommended. While these adapted guidelines are helpful, the data on best treatment modalities in AHI remain limited.

To enhance identification and management of persons with AHI, the Duke-UNC Acute HIV Infection Research Consortium was founded as a collaboration between Duke University and University of North Carolina, Chapel Hill (UNC). Our consortium previously reported results using a non-nucleoside reverse transcriptase inhibitor (NNRTI)-containing regimen with efavirenz, emtricitabine and tenofovir disoproxil fumarate (EFV/FTC/TDF) and a dual, NRTI-sparing regimen in acutely infected participants.^9–11^ Here, we present results with a co-formulated, once-daily treatment regimen started during AHI commonly used at the time of the study: elvitegravir, cobicistat, emtricitabine and tenofovir disoproxil fumarate (EVG/COBI/FTC/TDF or Stribild^®^). Our primary objective was to determine the virologic efficacy of this once-daily, fixed-dose combination (FDC) for participants with AHI as determined by the proportion of treated participants with HIV-1 RNA <200 and <50 copies/mL by treatment Weeks 24 and 48, respectively. We also compared virologic efficacy and time to suppression to a historical control cohort treated with FDC EFV/FTC/TDF during the period of acute infection.

## Materials and methods

### Study population

The study population included participants ≥18 years of age who met criteria for acute HIV diagnoses. The majority were referred from the North Carolina (NC) Screening and Tracing Active Transmission Program, a statewide screening programme for detection of AHI in publicly funded testing sites that used nucleic acid amplification (NAAT testing) of pooled HIV-1 antibody-negative samples and confirmed by subsequent HIV-1 antibody testing.^12–14^

This study was conducted in HIV clinics at Duke and UNC as a single arm, 48-week, open-label study assessing the efficacy of a once-daily, INSTI-based regimen using EVG/COBI/FTC/TDF (Stribild^®^) in participants newly diagnosed with AHI. The study population included men and women of diverse ethnicity and race. AHI was defined as a negative or indeterminant HIV-1 antibody, antigen or NAAT test within 30 days of study entry, plus any one of the following: (i) a detectable HIV nucleic acid in blood; (ii) a positive p24 antigen; (iii) a positive HIV antibody test according to standard criteria obtained within 45 days after the initial negative or indeterminate test described above; or (iv) a positive fourth generation HIV Ag/Ab combination assay and a negative HIV rapid test or negative or indeterminate Western Blot within 30 days of study entry. Exclusion criteria included the following: pregnancy, breast feeding, inability to commit to acceptable contraceptive methods to prevent pregnancy, known allergy to study drugs, difficulty swallowing capsules, inability to communicate with study personnel, active drug or alcohol dependence, recent acute hepatitis, severe acute illness, incarceration, recent/current administration of experimental therapy and use of immune modulating agents or contraindicated medications. Genotype and immunological assays were collected at baseline.

Enrolled study participants were followed for 96 weeks with study visits scheduled at Weeks 0 (enrolment), 2, 4, 8, 12, 16, 24, 36, 48, 60, 72, 84 and 96. FDC ELV/COBI/FTC/TDF was provided by the study for the first 48 weeks.

### Historical comparison cohort

We compared outcomes observed in the INSTI-based AHI cohort to those from an historical cohort of individuals who initiated treatment with FDC EFV/FTC/TDF in an earlier AHI study conducted by the Duke-UNC AHI Study consortium.^11^ Briefly, 90 persons with AHI (similarly defined) were included in a dual-centre, single-arm, open-label study of FDC EFV/FTC/TDF conducted between 2005 and 2011. Participants were followed for 96 weeks. Efficacy was assessed by the proportion of participants with HIV-1 RNA <200 copies/mL by Week 24 and HIV-1 RNA <50 copies/mL at Week 48.^10,11^

### Analysis

We used descriptive statistics [frequencies/proportions or medians/interquartile ranges (IQR)] to summarize demographics (age at diagnosis, sexual risk group and race/ethnicity) and clinical characteristics of participants enrolled in the INSTI and NNRTI AHI cohorts. Clinical characteristics of interest included HIV-1 RNA and CD4 cell count at enrolment, time from diagnosis to ART initiation, time to viral suppression (HIV-1 RNA <200 copies/mL and <50 copies/mL) and weight gain after treatment initiation. Efficacy analyses at Weeks 24 and 48 were different from previously published results^10^; as we excluded participants who were no longer on study, and the last available viral load was carried forward for participants who missed a given study visit. Differences between the two groups were assessed using Wilcoxon rank-sum tests for continuous variables and Fisher’s exact tests for categorical variables.

The time from ART initiation to viral suppression was estimated using Kaplan–Meier curves. Estimates were compared using log-rank tests. Participants lost to follow-up prior to Week 24 and participants without an available viral load at enrolment were excluded in the time to suppression analysis. Adverse events (AEs) among those enrolled in the current study were characterized using descriptive statistics. All analyses were performed using SAS v.9.4 (SAS Institute; Cary, NC).

The protocol was approved by both the Duke University and UNC Institutional Review Boards; written informed consent was obtained from all study participants.

## Results

Between September 2012 and April 2015, 33 participants with AHI were enrolled and started on EVG/COBI/FTC/TDF. Two participants were not included in the analysis: one participant withdrew after Week 8 due to moving and one participant withdrew immediately after enrolment due to lack of transportation, leaving 31 participants for the efficacy analysis at Week 24. For time to suppression analyses, another two participants were excluded since they did not have a viral load available at enrolment (leaving *n* = 29). Among 31 participants, the median age at diagnosis was 26 years (IQR 22–42). Most participants were African American (*n* = 19, 61%) and identified as men who have sex with men (MSM) (*n* = 22; 73%); seven participants were female (23%). Black MSM comprised almost half of the analysis population (*n* = 13; 43%) and were younger than other participants [median age of Black MSM was 24 (IQR 22–26) compared with 36 years (IQR: 24–46) among others]. Male participants were younger than female participants with a median age of 26 (IQR 22–34) and 37 years (IQR 23–44), respectively. Baseline demographic data were similar between participants enrolled in the two AHI studies (Table 1). Among the 31 participants in the INSTI cohort, 84% (*n* = 26) were retained through Week 48, and 61% (*n* = 19) through Week 96.

**Table 1.** Baseline demographics and clinical characteristics of acutely infected participants in INSTI cohort, compared with historical NNRTI cohort^11^

	EVG/COBI/FTC/TDF (*N* = 31) *N* (%)/median (IQR)	EFV/FTC/TDF (*N* = 90) *N* (%)/median (IQR)	*P*-value
Age (years)	26 (22–42)	28 (22–38)	0.97
Weight (kg), enrolment^a^	84 (74–94)	80 (70–87)	0.20
Transmission risk category^a^			
Female	7 (23)	11 (12)	0.24
Heterosexual male	1 (3)	9 (10)	
MSM	22 (73)	70 (78)	
Race/ethnicity			
White, non-Hispanic	10 (32)	36 (40)	0.48
White, Hispanic	2 (6)	2 (2)	
African American	19 (61)	52 (58)	
HIV viral load (copies/mL)			
Enrollment^a^	77 233 (16 727–1 043 734)	169 365 (37 706–750 000)	0.24
CD4 cell count (cells/mm^3^)			
Enrollment^a^	513 (397–715)	487 (319–651)	0.30
AHI diagnosis to ART start (days)	13 (10–22)	19 (14–25)	0.04
ART start to <200 copies/mL (days)^a^	26 (14–35)	64 (29–104)	<0.01
ART start to <50 copies/mL (days)^a^	54 (26–84)	99 (61–162)	<0.01

MSM, men who have sex with men; AHI, acute HIV infection; ART, antiretroviral; IQR, interquartile range.

^a^Missing transmission risk category: *n* = 1; missing enrolment weight: *n* = 5; missing enrolment HIV RNA level: *n* = 2; missing enrolment CD4 cell count: *n* = 3.

Median HIV-1 viral load at enrolment was 77 233 copies/mL (IQR: 16 727–1 043 734) and 169 365 copies/mL (37 706–750 000) among participants in the INSTI and NNRTI studies, respectively. The median CD4 cell count at enrolment among participants in INSTI and NNRTI studies was 513 cells/mm^3^ (IQR 397–715) and 487 cells/mm^3^ (IQR: 319–651), respectively. Enrolment HIV-1 RNA levels among participants in the INSTI study were as follows: 17% < 10 000 copies/mL; 38% between 10 000 and 99 999 copies/mL, 17% between 100 000 and 999 999 copies/mL, and 28% ≥1 000 000 copies/mL. No participant had baseline mutations conferring resistance to study treatment; genotypic testing was not available for one participant due to insufficient amplification.

At Week 24, HIV-1 viral suppression was similar between cohorts with 100% (*n* = 29/29) of those on the INSTI-based regimen with <200 copies/mL versus 99% (*n* = 85/86) on the NNRTI regimen. At Week 48, both groups had obtained 100% HIV-1 viral load suppression to <50 copies/mL (INSTI *n* = 26/26; NNRTI *n* = 84/84). More participants in the INSTI cohort had HIV-1 RNA levels <50 copies/mL at Week 16 [97% (*n* = 28/29) versus 72% (*n* = 62/86); *P* = 0.004] compared with those in the NNRTI cohort, but by Week 24 the results were not statistically different [97% (*n* = 28/29) versus 90% (*n* = 77/86); *P* = 0.45].

**Figure 1.**
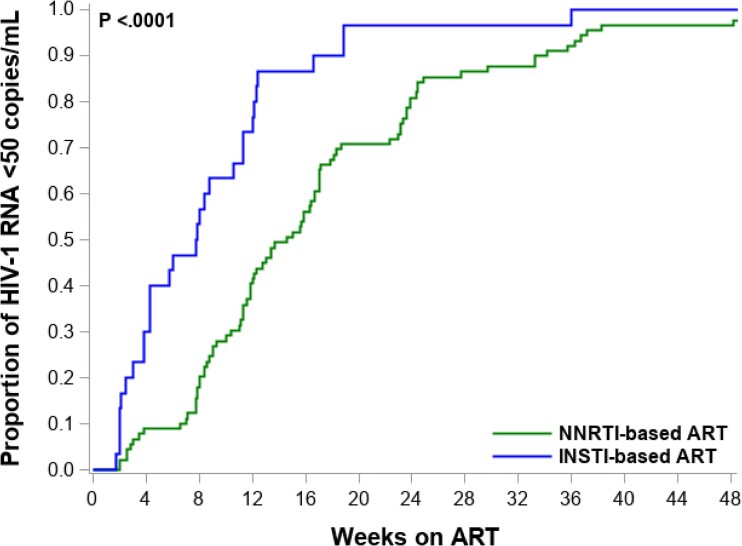
Cumulative incidence of viral suppression (HIV-1 viral load <50 copies/mL) among participants enrolled in INSTI-based ART and NNRTI-based ART.

Time from ART initiation to HIV-1 suppression (<50 copies/mL) was shorter in participants treated with INSTI-based ART versus NNRTI-based ART at a median of 54 days (IQR: 26–84) compared with 99 days, respectively (IQR: 61–162) (*P* < 0.01) Table 1; Figure 1. There was no significant difference in CD4 cell count recovery between the two ART regimens. The median change in CD4 count from baseline with INSTI-based ART was +200 cells/mm^3^ (IQR: 79–359) at Week 24 and +269 cells/mm^3^ (IQR: 113–462) at Week 48 compared with CD4 count increase on NNRTI-based ART of +161 cells/mm^3^ (IQR: 42–315) at Week 24 (*P* = 0.32) and +204 cells/mm^3^ at Week 48 (IQR: 77–399) (*P* = 0.51).

A total of 82 unique AEs were reported among 19 of 31 (61%) participants in the current INSTI cohort. The most commonly reported AEs were diarrhoea (7 events in 4 of 31 participants), headache (7 events in 5 of 31 participants) and postural hypertension (7 events in 7 of 31 participants). There were no study treatment-related serious AEs during the study.

**Figure 2.**
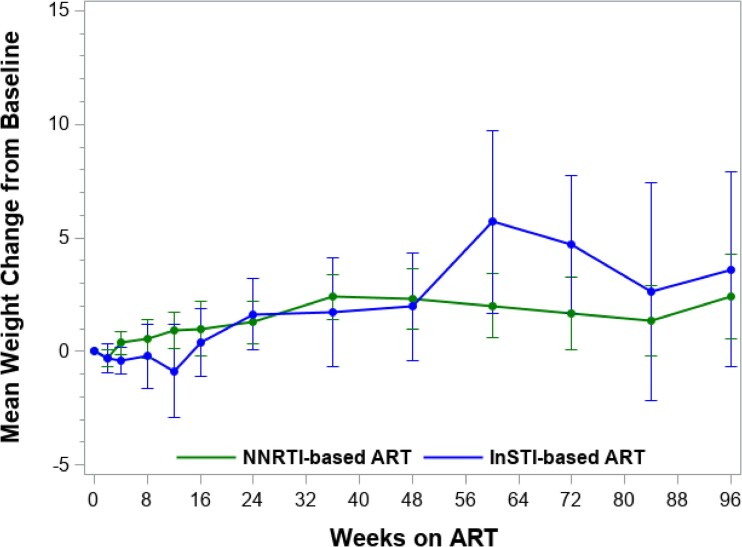
Mean change in weight and 95% confidence intervals from study baseline among participants enrolled in INSTI-based ART and NNRTI-based ART.

Among individuals with non-missing weight data, the mean weight change at Week 24 increased by +1.6 kg among INSTI participants (*n* = 28) and +1.3 kg among NNRTI participants (*n* = 77) (*P* = 0.58) Figure 2. By Weeks 48 and 96, the mean weight change remained similar between the two groups: +2.0 kg among INSTI participants (*n* = 25) and +2.3 kg among NNRTI participants (*n* = 73) (*P* = 0.99) at Week 48; +3.6 kg in INSTI participants (*n* = 19) and +2.4 kg in NNRTI participants (*n* = 58) and at Week 96 (*P* = 0.51).

## Discussion

Once-daily INSTI-based ART is the current standard of care in HIV treatment and is recommended in all major HIV treatment guidelines as initial therapy. Results from this study of elvitegravir-based ART for persons with acute HIV infection confirm the high efficacy of INSTI-based ART in this population. The demonstrated rapidity of HIV suppression, limiting the period of high infectiousness, represents a major advantage of INSTI-based ART for acute HIV infection. Since the study period, a TAF-containing version of this single tablet regimen, EVG/COBI/FTC/TAF (Genvoya^®^), in addition to similar single tablet regimens with an alternative INSTI component have been approved and are being widely used. In subsequent studies, the TAF-based regimen has been shown to have higher viral suppression rates comparatively (84% TAF versus 80% TDF at Week 144), driven mainly by fewer discontinuations in the TAF arm due to adverse effects.^15^ One may safely extrapolate that TAF-containing INSTI regimens would work equally well in our AHI cohort.^16^ Compared with data from our earlier study of NNRTI-based ART in AHI, the INSTI-based regimen demonstrated more rapid viral suppression and greater overall rates of HIV suppression compared with the EFV/FTC/TDF treatment regimen, with few AEs reported. While any debate over the recommendation to start ART during AHI has waned,^17^ our findings support the use of INSTI-based treatment for acute HIV infection.

Since this study was conducted, the ‘second generation’ INSTIs, DTG and BIC, have become preferred INSTIs for treatment of HIV and are recommended by the current DHHS Guidelines, including treatment for AHI.^8^ This recommendation is based on the absence of pharmacologic boosters in the newer INSTIs (reducing the risk of potential drug–drug interactions), as well as a higher barrier to resistance of these newer agents. At present, the rate of transmitted INSTI resistance remains low including in North Carolina.^18^ DHHS does not currently recommend testing for INSTI resistance in treatment-naïve patients, including those with AHI, unless the individual has a history of CAB-LA use for PrEP or INSTI use for post-exposure prophylaxis.^8^ As INSTI use expands, though, surveillance for INSTI resistance may become warranted.^19,20^ Additionally, there does not appear to be a benefit to using intensified regimens in early or acute HIV infection,^21,22^ supported by our findings of rapid suppression with an INSTI-based regimen.

In this study, treatment of acute HIV infection with an INSTI-based regimen resulted in more rapid viral suppression compared with AHI participants in an historical cohort treated with a NNRTI-based regimen. AEs were relatively uncommon in the INST-based cohort and did not lead to treatment discontinuation in any study participant, demonstrating another advantage of INSTI-based ART. By Week 96, both cohorts had similar weight gains, although greater among the INSTI cohort as demonstrated in other studies.^23^ Limitations of these data included the relatively small sample size and the higher baseline HIV-1 viral load observed in those treated with NNRTI-based ART.

While EVG/COBI/FTC/TDF has predominantly been replaced with other INSTI regimens, our results most likely can be extrapolated to more current regimens and demonstrate that INSTI-based regimens can effectively and safely treat persons with acute HIV.
